# The Role of Ras-Associated Protein 1 (Rap1) in Cancer: Bad Actor or Good Player?

**DOI:** 10.3390/biomedicines8090334

**Published:** 2020-09-07

**Authors:** Chin-King Looi, Ling-Wei Hii, Siew Ching Ngai, Chee-Onn Leong, Chun-Wai Mai

**Affiliations:** 1 School of Postgraduate Study, International Medical University, Bukit Jalil, Kuala Lumpur 57000, Malaysia; chin_king93@hotmail.com (C.-K.L.); lingweihii@imu.edu.my (L.-W.H.); 2 School of Pharmacy, International Medical University, Bukit Jalil, Kuala Lumpur 57000, Malaysia; cheeonn_leong@imu.edu.my; 3 School of Biosciences, Faculty of Science and Engineering, University of Nottingham Malaysia, Semenyih, Selangor 43500, Malaysia; Eunice.Ngai@nottingham.edu.my; 4 Centre for Cancer and Stem Cells Research, Institute for Research, Development, and Innovation (IRDI), International Medical University, Bukit Jalil, Kuala Lumpur 57000, Malaysia

**Keywords:** Rap1, Rap1GAP, tumor initiation, tumor progression

## Abstract

Metastasis is known as the most life-threatening event in cancer patients. In principle, the immune system can prevent tumor development. However, dysfunctional T cells may fail to eliminate the tumor cells effectively and provide additional survival advantages for tumor proliferation and metastasis. Constitutive activation of Ras-associated protein1 (Rap1) has not only led to T cell anergy, but also inhibited autophagy and supported cancer progression through various oncogenic events. Inhibition of Rap1 activity with its negative regulator, Rap1GAP, impairs tumor progression. However, active Rap1 reduces tumor invasion in some cancers, indicating that the pleiotropic effects of Rap1 signaling in cancers could be cancer-specific. All in all, targeting Rap1 signaling and its regulators could potentially control carcinogenesis, metastasis, chemoresistance and immune evasion. Rap1GAP could be a promising therapeutic target in combating cancer.

## 1. Introduction

Cancer has been identified as a major public health problem and the leading cause of death in developed and developing countries. The number of cancer deaths are on the rise globally. There is an expected 45% increase in cancer incidence from 2010 to 2030. The high mortality cancers, such as pancreas, liver and lung, will remain as cancers with poor prognosis [1,2]. The uncontrolled growth of malignant cells poses the ability to spread to other tissues or organs beyond the primary tumor site, leading to the event of metastasis. The loss of cell–cell adhesion allows the dissociation of tumor cells from the primary tumor site. The alteration of cell-matrix adhesion provides tumor cells an opportunity to invade surrounding stroma, ultimately resulting in tumor invasion [3]. The gain of mesenchymal characteristics is called epithelial mesenchymal transition (EMT) [4,5,6]. The ability of tumor cells to undergo EMT has not only contributed to tumor invasion, but also drug resistance and tumor survival in the primary tumor microenvironment (TME) upon systemic dissemination [6]. The tumor cells may reverse EMT through mesenchymal–epithelial transition (MET) after dissemination, with an enhanced ability to undergo metastatic outgrowth [7,8]. Hence, both EMT and MET may enhance tumor initiation and tumor invasion.

During each step of the metastatic cascade, the highly immunogenic tumor cells may be recognized and eliminated by the host immune system [9,10,11]. However, until today, the complex interaction between cancer cells and immune cells is yet to be clearly defined. In general, antigen presenting cells (APCs) such as dendritic cells (DCs) phagocytose tumor antigens, and the fragmented antigens are then presented by major histocompatibility complex (MHC) class I and II to cytotoxic T lymphocytes (CTLs) and cluster of differentiation (CD)4^+^ T cells, respectively. Activated T cells infiltrate into tumors in order to kill the cancer cells [12,13]. Cellular adhesion molecules such as integrins and the immunoglobulin superfamily may initiate T cell adhesion to APCs [14].

**Figure 1:**
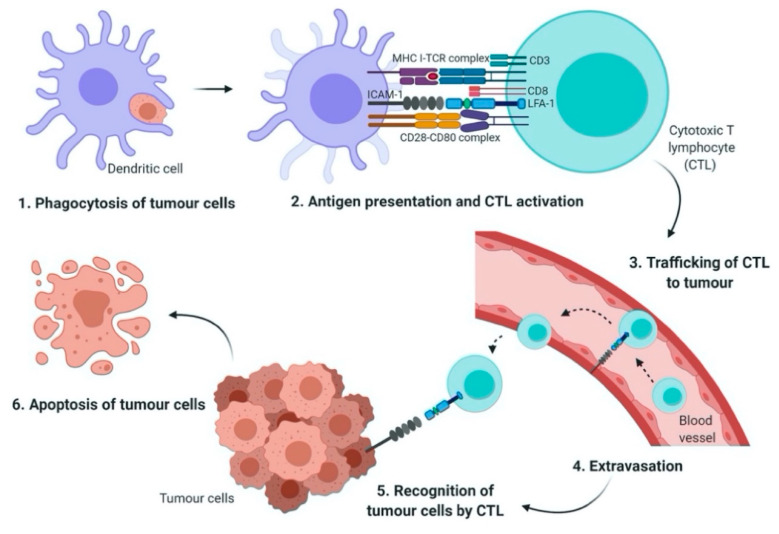
The role of LFA-1/ICAM-1 in T cell activation and tumor eradication. Antigen-presenting cells (APC) such as dendritic cells (DC) phagocytose tumor cells and then present the tumor antigens via MHC class I molecules (Step 1). Cytotoxic T lymphocyte (CTL) requires two signals to become fully activated; first, the interaction of T cell receptor (TCR) with MHC-peptide complex, second, co-stimulatory signals generated through the interaction of CD28 on CTL with CD80 on APC. The adhesion of CTL to the DC is mediated by leukocyte function-associated antigen 1/ intercellular adhesion molecules 1 (LFA-1/ICAM-1) interaction (Step 2). The activated CTL is then travelled from the lymph node and enters the blood stream (Step 3). Tumor cell extravasation involves the adhesion of LFA-1 with ICAM-1 which is expressed on the endothelial cells (Step 4). Finally, in the tumor microenvironment, CTL recognizes and binds to the target tumor cells via formation of immunological synapse and LFA-1/ICAM-1 interaction (Step 5). This in turn, results in the apoptosis of tumor cells (Step 6).

Interestingly, integrin may mediate the infiltration of immune cells to the tumor site, the activation and proliferation of effector cells, and the formation of immunological synapse between immune cells and tumor cells [14]. The leukocyte function-associated antigen-1 (LFA-1) is a member of the β2 integrin family of adhesion receptor. It is also the predominant integrin in lymphocytes. It is an important component in T cell trafficking and extravasation [15] by binding to its ligand, the intercellular adhesion molecules-1 (ICAM-1) [14]. The LFA-1/ICAM-1 activates the formation of the immunological synapse via cell–cell interactions between immune effector cells and tumor cells (Figure 1). Tumor cells may gain resistance towards immune cell-mediated cytotoxicity when LFA-1/ICAM-1 is disrupted by matrix metalloproteinases 9 (MMP 9) [16].

The integrin is not constitutively activated. Instead, the adhesiveness of LFA-1 in T cells is tightly regulated. Initially, normal resting T cells express a low affinity or inactive form of LFA-1 and convert into a high affinity or active form, and subsequently promote adhesive interaction with ICAM-1 after stimulation with external stimuli [17]. The small GTPase Ras-associated protein 1 (Rap1) is one of the main components of inside-out signaling cascades that plays a critical role in activating integrin [14,15]. Indeed, Rap1 signaling has been shown to play a role as a potent immunological regulator in modulating LFA-1-mediated adhesion in Jurkat T cells [18]. Activation of Rap1 in the T cells rapidly increased LFA-1 affinity, while overexpression of dominant negative form of Rap1 (Rap1N17) or signal-induced proliferation-associated gene-1 (SPA-1) completely inhibited LFA-1 activation [19] and reduced adhesiveness and interleukin (IL)-2 production in Jurkat T cells [18]. Conversely, overexpression of Rap1 enhanced T cell activation and augmented IL-2 production [18]. In addition, in vivo study showed that increased active Rap1 in T lymphocytes was associated with enhanced T cell receptor (TCR)-mediated response and increased integrin activation [20]. Hence, these findings clearly indicate that Rap1 plays a crucial role in mediating integrin activation in T cells via inside-out signaling cascades.

Nonetheless, Rap1 activation may need to be downregulated after the initiation of immunological synapse formation in order to achieve its optimal T cell activation. A constitutively activated Rap1-GTP may inhibit TCR- and CD28-mediated IL-2 transcription significantly [21]. Another study showed that constitutive activation of Rap1 was associated with reduced IL-2 production and an increase of activation-induced cell death (AICD) of T cells or T cell anergy [18]. Besides that, due to the defective downregulation of Rap1-GTP, the naïve T cells in SPA-1-deficient mice was found to be in an anergic state [22]. As a result, the precise control of Rap1 activation is critical for T cell activation. The dysregulation of Rap1 signal has been evidenced in tumor invasion and metastasis.

## 2. Biology and Regulation of Rap1

**Figure 2:**
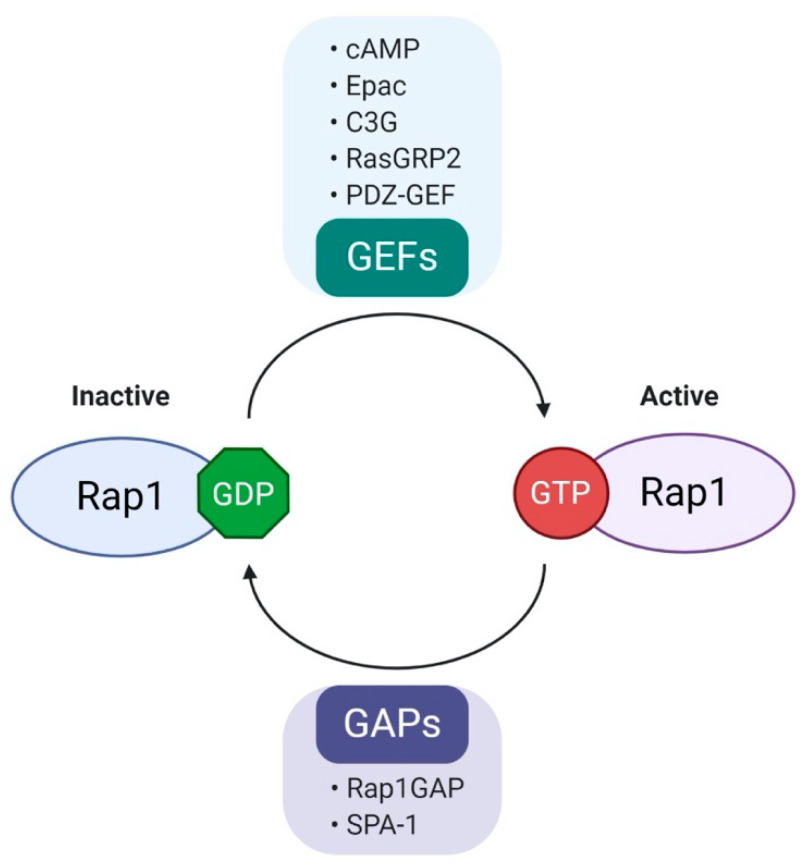
Regulation of Rap1 activity. Rap1, a small GTPase that acts as a molecular switch and cycles between GDP (inactive state) and GTP (active state). Rap1 activity is positively regulated by guanine nucleotide exchange factors (GEFs) and negatively regulated by GTPase activation proteins (GAPs). Various GEFs and GAPs are involved in the regulation of Rap1 activity. (cAMP, cyclic AMP; Epac, exchange protein activated by cAMP; C3G, Crk SH3-binding guanine nucleotide-releasing protein; RasGRP2, Ras guanyl-releasing protein 2; PDZ-GEF, PDZ- guanine nucleotide exchange factors; Rap1GAP, Rap1 GTPase-activating protein; SPA-1, signal-induced proliferation-associated gene-1.).

Rap1 is a member of the Ras-like small GTP binding protein family. Rap1 can bind to either guanosine triphosphate (GTP) or guanosine diphosphate (GDP). Rap1 activity is positively regulated by guanine nucleotide exchange factors (GEFs) and negatively regulated by GTPase-activating proteins (GAPs) [23]. GEFs promote dissociation of GDP, allowing loading of GTP to Rap1, resulting in the active form of GTP-bound Rap1 (Rap1-GTP). Conversely, Rap1-GTP is effectively hydrolyzed into inactive GDP-bound Rap1 (Rap1-GDP) in the presence of GAPs [23]. The well-characterized GEF family includes C3G (RapGEF1), RasGRP2, PDZ-GEF, cAMP and Epac [24]. In contrast to the diverse types of GEFs, there are only two groups of GAPs, Rap1GAP and SPA-1 [17]. The regulation of biological activity of Rap1 is summarized in Figure 2.

Rap1 has two isoforms; Rap1A and Rap1B, sharing 95% amino acid sequence identity [25]. Although some studies emphasized the roles of Rap1A and Rap1B in tumor progression, little has been reported on the functional differences between these two isoforms. In prostate [26] and head and neck squamous cell carcinoma (HNSCC) [27], Rap1A has been shown to promote invasion and migration of tumor cells. Few cancer studies have shown that expression of Rap1B was associated with an increased ovarian cancer migration [28] and esophageal cancer invasion [29]. Furthermore, the upregulation of Rap1 is associated with aggressive phenotypes of malignancy [30,31]. Collectively, it suggests that both Rap1 isoforms may have an overlapping role in tumor progression. 

Rap1 was first discovered as a suppressor of Ras transformation in NIH 3T3 cells [32], in which Rap1 can antagonize Ras activity by competing with Ras for Raf-1, trapping Ras effectors in an inactive complex [33]. Rap1 and Ras share similar binding partners, such as Raf-1, B-Raf, Ral-GDS and phosphoinositol-3 kinase (PI3K). It is important to highlight that the binding partner has great impacts on the activation of Rap1. PI3K itself may modulate cancer progression [34,35]. The binding of Raf-1 to Rap1-GTP leads to Rap1 inactivation, while the binding of B-Raf to Rap1-GTP will promote Rap1 activity [36]. Besides that, Rap1 and Ras share a high degree of sequence identity (53%) at the amino acid level [23]. However, earlier studies have indicated that both Rap1 and Ras have significantly different affinity towards the downstream effectors, such as Raf-1 and RalGDS [37]. In addition, the activation of Rap1 and Ras is shown to occur at a distinct location. Rap1 converts to the active GTP-bound form in the perinuclear region of the cells, whereas Ras activation takes place in the plasma membrane [38]. Together, despite sharing a strong homology in amino acid sequence, Rap1 and Ras have different affinities towards the downstream effectors at a distinct subcellular location.

Initial functional studies in *Drosophila* testis had demonstrated that Rap1 signaling is involved in morphogenesis by facilitating cadherin-mediated cell adhesion [39] and adherens junction positioning [40]. The active Rap1 may promote cell polarity and migration by coordinating the cytoskeleton reorganization [41,42]. Since normal tissues require tightly regulated homeostasis of cell–cell and cell–extracellular matrix signaling, any aberrations in these processes may induce tumorigenesis [43]. As such, it is unsurprising to find that the aberrant overactivation of Rap1 in human epithelial cells may induce tumor formation and progression. It certainly warrants further investigation to elucidate the role of Rap1 in cancer formation and progression.

## 3. Rap1 and Rap1GAP in Cancer

**Figure 3:**
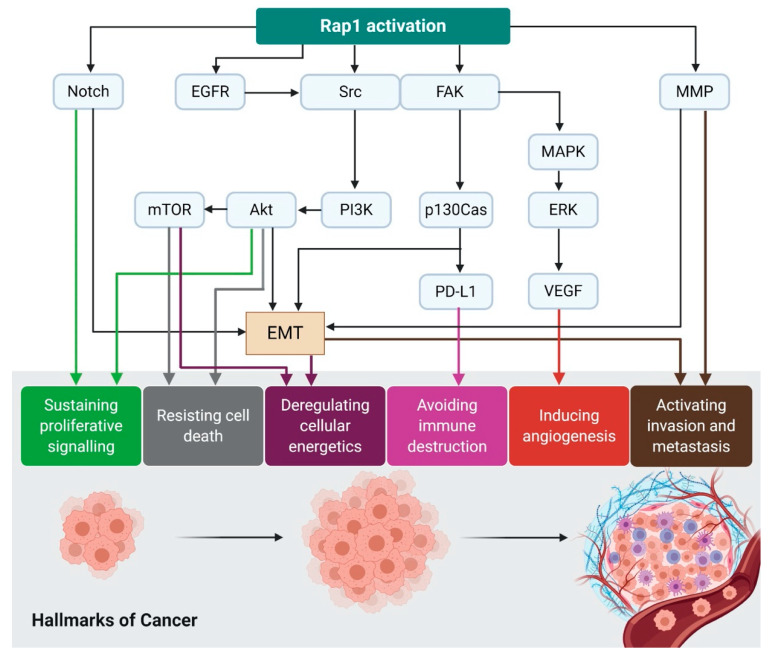
Rap1 activation contributes to the acquisition of cancer hallmarks. Rap1 plays a diverse role in tumor initiation and progression. For instance, Rap1 activation induces tumor initiation and epithelial–mesenchymal transition (EMT) via Notch signaling. EGFR and Src/FAK may be stimulated by the activated Rap1, leading to integrin-mediated cell adhesion in cancer. The adhesion of integrins to the extracellular matrix (ECM) is an essential step for tumor cell invasion and metastasis. Src may activate MAPK/ERK to increase VEGF expression levels in the tumor cells, ultimately leading to angiogenesis. Src may also induce p130Cas and PD-L1 expression in the tumor cells. The upregulation of MMP during Rap1 activation may trigger EMT in cancer and thus enhancing invasiveness and metastasis. Therefore, Rap1 has rendered itself to be an attractive therapeutic target in cancer treatment.

The emergence of significant evidence has proven the involvement of Rap1 in tumorigenesis in various model systems. As shown in Figure 3, Rap1 activation confers several hallmarks of cancer through ERK, MAPK, and Src/FAK [44]. The activated Rap1 is highly expressed in cancerous cells compared to non-cancerous cells. For example, in breast epithelial cells, an increased glucose uptake associates with an upregulation of integrin through Rap1, leading to acquisition of malignant phenotype in non-cancerous breast cells [45]. On the contrary, Rap1GAP, the negative regulator of Rap1 activity, is always found to be dysfunctional or downregulated in many malignancies, leading to an increase of Rap1-GTP. In some instances, Rap1GAP expression is further downregulated in cancers, suggesting that its depletion contributes to tumor progression [46,47]. Additionally, a re-expression of Rap1GAP was associated with a reduction in tumor size [47]. Interestingly, Rap1 (Table 1) and Rap1GAP (Table 2) demonstrated contrasting effects on a tumor. Very little is known about the definitive role of Rap1GAP in cancers. It remains uncertain whether Rap1 signaling will positively or negatively regulate tumor progression. Therefore, further research is required to delineate its roles in cancers.

Most of the published studies suggest that Rap1GAP is frequently downregulated in various tumors; however, its precise mechanisms may vary in different cell types. The silencing or inactivation of Rap1GAP could be due to the loss of heterozygosity (LOH), methylation of promoter, or gene mutation. Pancreatic cancer [59] and thyroid cancer studies [60] demonstrate that the loss of Rap1GAP activity was associated with a high frequency of LOH of the Rap1GAP gene [59]. LOH for Rap1GAP gene induced tumor growth, invasion, and survival in pancreatic cancer [59]. In melanoma [57] and renal cell carcinoma [61], Rap1GAP downregulation was mediated through promoter hypermethylation. On the other hand, Rap1GAP gene was silenced by Enhancer of Zeste Homolog 2 (EZH2), a histone methyltransferase, which facilitates hypermethylation of Rap1GAP promoter in HNSCC [62].

### 3.1. Rap1 Regulates Autophagy and Cancer Metabolism via mTORC1

Autophagy is an important self-degradative process which involves degradation of damaged cellular components by lysosomes, disposal of intracellular aggregates, and the elimination of intracellular pathogens [63,64]. This process is essential in homeostasis [65]. Autophagy can be induced by environmental stresses such as hypoxia, pathogen infection, nutrient starvation, oxidative stress, or DNA damage [66,67]. Importantly, the dysregulation or dysfunction of autophagy have been implicated in tumorigenesis [68,69]. In addition, stress-induced autophagy in ovarian tumor cells was shown to be associated with an increased drug resistance, prolonged tumor cell survival, and promoted tumor dormancy [70]. Furthermore, due to insufficient blood supply, the rapidly multiplying tumor cells often lead to the development of hypoxic and nutrient-deprived TME. Therefore, tumor cells may metabolically reprogram and engage autophagy processes in order to forestall senescence or death [67]. While autophagy-induced senescence in tumor cells may limit growth, autophagy-induced senescence in the tumor stroma may promote tumor growth and progression [67].

The mammalian target of rapamycin (mTOR) is one of the main signaling integrators and regulators of autophagy [67]. It forms two different types of protein complexes, which are defined by the proteins to which it is associated. The presence of Raptor, mLST8/GβL, Deptor and Pras40 form mTOR complex 1 (mTORC1), while the presence of Rictor, GβL, Protor, Deptor and mSin form mTORC2 [71]. mTORC1 acts as a master sensor of metabolites that responds to growth factors, glucose, amino acids, oxygen level; whereas mTORC2 only responds to growth factors [67,71]. Furthermore, mTORC1 has been implicated in autophagy regulation of tumor cells in response to nutrient signals. It is reported to be mostly dysfunctional among cancer cells [67,72]. Under nutrient-rich conditions, mTORC1 is translocated to the lysosome where it is activated to inhibit autophagy. This in turn promotes the biosynthesis of proteins, lipids, and nucleotides [72,73]. However, the inhibition of mTORC1 activity due to nutrient deprivation remains poorly understood.

A recent study established that amino acid levels can alter the signaling of Rap1 pathway [72]. Rap1GTP was found to inhibit the translocation of mTORC1 and reduce overall lysosome abundance when the availability of amino acids is limited [72]. Rap1 expression inhibits mTORC1 activation during low levels of amino acids by limiting the expansion of lysosomes, and consequently, Rap1 depletion leads to mTORC1 hyperactivity. However, constitutive activation of Src (which can be promoted by Rap1 activation [74]) has been shown to sustain mTORC1 activity and promotes cell survival under nutrient deprivation conditions [75]. Combinatorial treatment with Src inhibitor (dasatinib) and mTORC1 inhibitor (rapamycin) reduced tumor growth [76]. Hence, these controversial results indicate the need of further studies to validate the role of Rap1 in regulating mTORC1 activity.

### 3.2. Rap1 Signalling Promotes Integrin-Mediated Cell Adhesion

Cell adhesion plays an integral role in regulating cell differentiation, migration and survival [77]. Integrin and cadherin are the two well-characterized transmembrane adhesion receptors with the ability to influence cell growth and cell survival [78]. However, integrin and cadherin display an antagonistic relationship in cancer and metastases. Such a relationship determines whether cells maintain connection with neighboring cells or loss of contact. For instance, the ligation of integrin to collagen in ovarian carcinoma cells resulted in the downregulation of E-cadherin through MMP9 [79].

**Figure 4:**
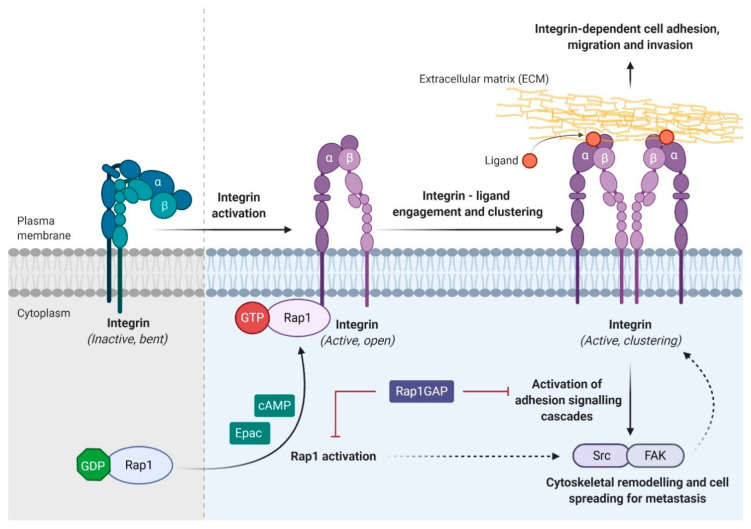
Rap1 regulates integrin-dependent biological processes in cancers. Integrin, the main cell adhesion receptor for the extracellular matrix (ECM), exists in different conformational states, and thus leads to the difference in its receptor affinity. An inactivated integrin (bent and closed) is associated with a low affinity for ECM ligand, whereas the active form (open) integrin is capable of binding to the ligand and clustering on the plasma membrane (adhesion). Integrin adhesion activates signaling pathway such as Src/FAK to promote cytoskeletal remodeling, tumor migration, invasion, and metastasis. Integrin activation can be inhibited by Rap1GAP directly or indirectly. Solid arrow indicates direct causative effect while dotted arrow indicates indirect causative effect.

Rap1 signaling has been implicated in the regulation of both integrin- and E-cadherin activity. Rap1 plays an essential role in various integrin-dependent biological processes (Figure 4), such as cell adhesion [80], migration of leukocytes and lymphocytes [19,81], immunological synapse formation [18], platelet adhesion and aggregation [82]. It has been well documented that Rap1 activation upregulates integrin expression while the inactivation of Rap1 by Rap1GAP markedly inhibits integrin-mediated migration and invasion [26]. Early study has revealed that overexpression of SPA-1, a GAP protein in promyelocytic 32D cells, was associated with abolished cell adhesion and inactivation of Rap1 [83], suggesting that Rap1-GTP is required for cell adhesion, and it is negatively regulated by GAP proteins. Another study reported a similar finding, showing that Rap1GAP abolished cAMP- and Epac-induced cell adhesion in ovarian carcinoma cells [84]. Epac1 is a cAMP-activated GEF that promotes cell adhesion and cell spreading through Rap1 activation [85]. Stimulation of melanoma cells using 8CPT-2Me-cAMP, a novel cAMP analogue, can induce Rap1 activation [74]. Rap1 was found to be translocated from cytoplasm to the plasma membrane, particularly in the protrusion of membranes undergoing spreading [74]. These results indicate that active Rap1 could induce integrin activation and consequently increase cell migration.

An early study of integrin-mediated adhesion in fibroblastic cells showed that the integrin adhesion is mediated by steroid receptor coactivator (Src) family through regulation of focal adhesion proteins and Rap1 activation [86]. Src kinase activity is required for tumor invasion and migration as it controls integrin adhesion turnover [87,88]. In melanoma, upregulation of Rap1 activity is associated with increased Src phosphorylation and integrin ligand-binding capacity, ultimately promoting integrin activation [74]. The use of active Rap1 (Rap1 12V) was shown to inhibit the effect of Csk, the negative regulator of Src [86]. In addition, the upregulation of Rap1GAP in thyroid carcinoma cells impaired cytoskeletal remodeling and migration via Src/FAK signaling [89]. In pancreatic cancer, epidermal growth factor receptor (EGFR) induced Src activation, followed by the phosphorylation of p130Cas/BCAR1 [52]. Rap1 activation was required for pancreatic cancer cell migration and EGFR-mediated metastasis [52]. These results clearly showed active Rap1 acts as an important modulator of Src signaling in the regulation of cell adhesion and integrin activation.

### 3.3. Rap1 Promotes EMT via Downregulation of E-cadherin Expression

**Figure 5:**
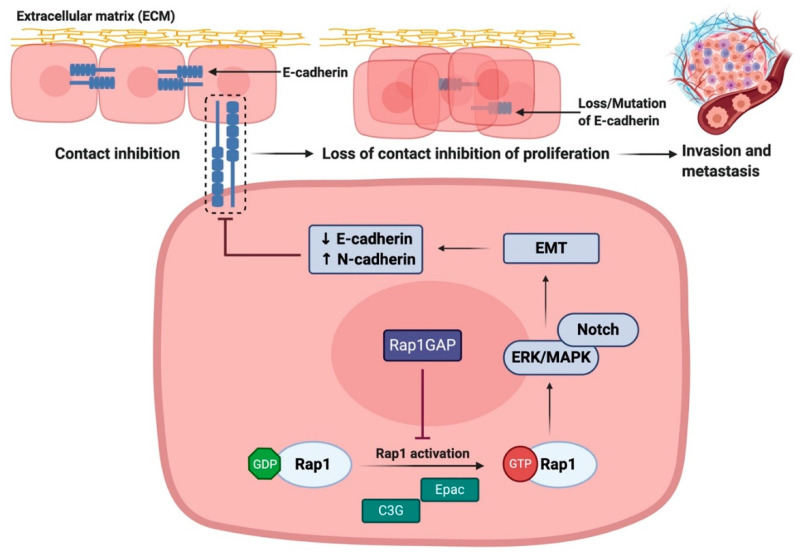
**Rap1 promotes tumor progression via disruption of E-cadherin-mediated cell adhesion.** Rap1 activation leads to the reduction in E-cadherin expression, resulting in the loss of epithelial cell–cell adhesive junctions and contact inhibition of proliferation. During the restoration of Rap1GAP expression in tumor cells, Rap1 will be inactivated and thus inhibiting tumor progression. ↑ indicates upregulation of the pathway while ↓ indicates downregulation of the pathway.

Rap1 has been reported to serve an important role in regulating cadherin expression (Figure 5). The downregulation of Rap1GAP was frequently associated in tumor cell lines which underwent epithelial-to-mesenchymal transition (EMT) [46,56]. A common feature of EMT is the reduction of E-cadherin expression, resulting in the loss of epithelial cell–cell adhesive junctions and contact inhibition of proliferation [90,91,92]. Recent study showed that upregulation of Rap1GAP was positively associated with the expression of E-cadherin in gastric cancer and colorectal cancer [46,56]. Mechanistic study showed that Rap1A enhanced the expression of EMT markers via activation of ERK, MAPK, and Notch signaling pathway [93]. Treatment with ERK inhibitor reversed the tumorigenic effect of Rap1, as evidenced by reduction in EMT markers expression and tumor migration [93]. Other studies have also shown that the depletion of Rap1GAP in breast cancer led to reduction in E-cadherin levels as well as ERK activation that is associated with enhanced tumor invasiveness [44].

In contrast, restoring Rap1GAP expression in tumor cells not only inhibits invasion and migration, but also inhibits anchorage-dependent proliferation [94]. Patients with higher combination of Rap1GAP and E-cadherin levels had better overall survival compared to patients with weak or no expression [58]. Collectively, these results suggest that the downregulation of Rap1GAP contributes to aggressive phenotypes of tumor, reduces E-cadherin expression, and increases expression of EMT features. Unfortunately, the disruption in the balance of Rap1 signaling as Rap1GAP may fail to perform as the negative regulator of Rap1 activity. As such, Rap1GAP and Rap1 may act as potential prognostic markers and novel targets of EMT and metastasis in cancers.

### 3.4. Rap1 Promotes Angiogenesis in Cancer

An imbalance between expression of angiogenic factors and angiogenic inhibitors is known to promote angiogenesis in cancers [95]. The ERK/MAPK signaling pathway enhances the transcription and expression of angiogenic factors, such as vascular endothelial growth factor (VEGF). ERK/MAPK induces angiogenesis in the tumor cells. In addition, VEGF also activates integrins (αVβ3, αVβ5, α5β1, and α2β1) and enhances cell adhesion and migration of endothelial cells to vitronectin [96]. The overexpression of Rap1GAP inhibited the angiogenic sprouting and tube-forming activity of endothelial cells [97]. VEGF increased Rap1 activity in order to promote angiogenesis in an integrin-dependent manner. The use of β1 integrin antibody was shown to inhibit constitutive active Rap1-induced angiogenesis in endothelial cells [97]. Lakshmikanthan and coworkers also demonstrated that VEGFR2 enhanced angiogenic response in endothelial cells via Rap1 activation [98]. Taken together, Rap1 is required for angiogenesis during development, consistent with the role of Rap1 in regulating integrin-mediated adhesion, which is essential for tubular structure formation.

### 3.5. Rap1 Signaling Promotes Cancer Cell Proliferation and Survival

Rap1 signaling has been implicated in the regulation of cell proliferation and migration in different cancers. The downregulation of Rap1GAP has been shown to transform breast ductal carcinoma in situ (DCIS) into an invasive ductal cancer (IDC) [44]. Retroviral silencing of Rap1GAP greatly enhanced ERK/MAPK phosphorylation and activation, induced extensive cytoskeletal reorganization and expression of EMT features, and enhanced invasion in DCIS cells [44]. Enforced re-expression of Rap1GAP in DCIS cells resulted in decreased Rap1 activation and a reversed mesenchymal to epithelial phenotype [44]. This study is in agreement with a previous study correlating upregulation of Rap1A with enhanced EMT markers expression and ERK activation in ovarian cancer cells. Conversely, the use of the ERK inhibitor U0126 inhibited ERK activation and reversed the effects of Rap1A [93]. These data build upon previous work in human umbilical vein endothelial cells (HUVECs), where similar effects were observed when pre-treatment of HUVECs with an ERK inhibitor, PD98059, not only reduced ERK phosphorylation, but also significantly inhibited cell proliferation [99]. These findings are in line with previous findings where overexpression of Rap1GAP in melanoma cells inhibited Rap1 activation and ERK phosphorylation, resulting in the reduction of tumor proliferation and survival [57].

### 3.6. Rap1 Signaling Regulates Matrix Metalloproteinases (MMPs)-Dependent Tumor Invasion and Metastasis

During cancer development, MMP participates in the degradation of the vascular basement membrane and extracellular matrix (ECM), promoting tumor neovascularization, and reducing cell adhesion [100]. In epithelial ovarian cancer, C3G (Rap1GEF1)-induced Rap1 activation promoted the secretion of MMP2 and MMP9. This in turn promoted the invasion and metastatic spread of tumor cells [101]. A downregulation of Rap1GAP in colorectal cancer patients was associated with increased MMP9 expression and poorer survival rates [56,102]. This result is in line with a study on gastric cancer, indicating that downregulation of Rap1GAP in patients significantly correlated with an upregulation of MMP9 with a poor prognosis [103]. In fact, it has been shown that expression of MMP9 alone is sufficient to cause downregulation of E-cadherin in kidney tubular epithelial cells and ovarian cancer [104,105]. Therefore, the downregulation of Rap1GAP expression is correlated with a poor prognosis in cancer. The inhibition of MMP secretion in prostate cancer cells further reduced Rap1-mediated adhesion, confirming the role of Rap1 in tumor cell invasion through integrin and MMP [26].

Remarkably, increased Rap1GAP expression in leukemia enhanced the invasion ability of leukemic cells via the upregulation of MMP9 expression [55]. In HNSCC, overexpression of Rap1GAP also increased the invasiveness and migration but not the proliferation of SCC cells. Its tumor invasiveness was blocked by MMP inhibitor [54], suggesting that Rap1GAP inhibits proliferation but enhances the invasive potential of SCC via MMP secretion. In contrast with another study in HNSCC, overexpression of Rap1-GTP facilitated tumor invasion through the expression of MMP7 [27]. This expression was resulted from the Rap1-GTP-induced nuclear translocalization of β-catenin, which in turn triggered the transcription of MMP7 to degrade the basement membrane. In fact, immunohistochemical studies of tissues from HNSCC patients revealed that the overexpression of active Rap1 was positively correlated with β-catenin expression and was associated with more advanced stages [27]. In contrast, active Rap1 can inhibit tumor invasion by inhibiting MMP2 and MMP9 [54,55]. However, the contradictory role of Rap1 in modulating MMP expression remains unclear. Thus, further studies are warranted to investigate the relationship between Rap1 and MMP in cancer.

### 3.7. Rap1 Signaling Mediates Chemoresistance and Immune Evasion in Cancer

The overexpression of Rap1GAP in pancreatic cancer cells enhances the basal apoptotic rates and increases drug sensitivity such as 5-fluorouracil (5-FU) and etoposide [59]. These results suggest that the downregulation of Rap1GAP in pancreatic cancer cells may protect the cells from drug-induced apoptosis.

The downregulation of E-cadherin via Rap1 activation may promote EMT in cancers. A recent study showed that the expression of EMT markers was positively correlated with PD-L1 expression in various types of cancers [106]. The EMT transcription factor, ZEB1, may promote lung cancer metastasis by modulating PD-L1 expression [107]. The silencing of ZEB1 in pancreatic cancer may also induce EMT conversion and apoptosis in chemoresistant cell lines [108]. Furthermore, the binding of Crk adaptor proteins to p130Cas or GTPases such as Rap1 promotes EMT, PD-L1 expression and immune evasion in breast cancer. In Crk knockout mice, the tumors exhibited enhanced CTL-mediated immune response, reduced PD-L1 expression and reversal of ZEB1-induced EMT [109]. These results collectively indicate that Rap1 plays an important role in chemoresistance and tumor immune evasion, despite further research being warranted to elucidate the underlying mechanisms.

## 4. Therapeutic Potential of Rap1 Signaling in Cancers

Despite the therapeutic advancement, it is sad to see that cancer mortality remains high. The search for new therapeutic approaches, novel compounds or combinatory agents [110,111,112] is still on-going. The challenge lies in the identification of a specific druggable target in cancers. Gene profiling data indicate that Rap1GAP levels are significantly downregulated in various malignancies. Immunohistochemical staining revealed that Rap1GAP expression is greatly reduced in poorly differentiated pancreatic cancer as compared to normal pancreatic tissues or chronic pancreatitis [59]. The same findings have also been reported in thyroid cancer [113]. Both in vivo and in vitro studies clearly showed that induced expression of Rap1GAP was associated with the decreased of pancreatic cancer growth, motility and invasiveness [59]. These findings suggest the potential role of Rap1GAP as a tumor suppressor. In colorectal, gastric and endometrial cancers, clinical studies have demonstrated that the loss of Rap1GAP expression was correlated with negative clinicopathological outcomes, such as invasion depth, lymph node metastasis, advanced TNM stages and poor overall survival (OS) as determined by Kaplan–Meier survival analyses [46,56]. Hence, these results clearly indicated that Rap1GAP may be used to target and identify patients who have a higher risk of recurrence and mortality, and personalized treatment can be given to improve patients’ clinical outcomes. The literature as above mentioned support a strong association between Rap1GAP and the development of aggressive malignancies. Therefore, Rap1GAP could serve as a therapeutic target in cancer therapy.

Rescued expression of Rap1GAP in renal cell carcinoma cell lines using a demethylating drug, decitabine (5-azadC), resulted in a significant reduction in tumor invasiveness [61]. Another compound, GGTI-298, a geranylgeranyltransferase I inhibitor, was shown to act as a Rap1 inhibitor that reduced migration of MCF7 breast cancer cells in a scratch-wound assay [51]. In pancreatic cancer, the use of a novel Epac-specific inhibitor (ESI-09), specifically inhibited Epac1-mediated Rap1 activation, ultimately resulting in impaired migration and invasion capability of cancer cells [114]. Unfortunately, ESI-09 has been found to show lack of isoform selectivity [115,116]. On the other hand, the administration of farnesylthiosalicylic acid (FTS) or FTS-amide (FTS-A) into mice with inflammatory disorders was reported to cause reduction in Rap1 activity and contact sensitivity reaction. FTS has been identified as a potential Ras inhibitor that was found to inhibit both farnesylation and geranylgeranylation of Ras. Surprisingly, the efficacy of FTS-A in Rap1 inhibition was better than in Ras inhibition, suggesting that Rap1 may function as a potential therapeutic target for FTS [117].

However, the design of inhibitors or small molecules with high specificity and yet minimal adverse effects that act to disrupt the tumor-promoting function of GTPases is particularly challenging. For example, previous study on the combination of FTS with GGTI inhibitor was shown to enhance apoptosis in vitro, however, it caused sudden death in mice [118]. Hence, the number of inhibitors identified for Rap1 are limited and have not progressed into clinical trials thus far. The inhibitors that target the small GTPases or indirect strategies involving GEF inhibitors or GAP activators were reported to have promising results in laboratory studies, but not during clinical trials [119]. In recent years, a combined structure-based virtual screening and high throughput screening utilizing fluorescent guanine nucleotide exchange assays discovered SOS1 inhibitor, a Ras GEF enzyme [120]. This unique screening strategy might potentially be used to discover other potential inhibitors targeting Rap GEFs in the future. Taking all these into account, Rap1GAP holds the high potential to serve as a novel target for therapeutic intervention. However, we are still far from achieving the identification and development of a specific Rap1 and/or GEF inhibitor as well as GAP activator. Hence, more efforts should be put into the screening of potential inhibitors with enhanced specificity and reduced toxicity in bridging the gap from bench to bedside.

## 5. Conclusions and Future Perspectives

Extensive and intensive studies have revealed the diverse biological activities of Rap1 and its association with increased malignancy. The Rap1 signaling is clearly shown to play a role in tumor promotion, through the activation of other signaling pathways such as MAPK/ERK, Src/FAK, or other novel pathways which have not been elucidated. While reduced expression of Rap1GAP is found in most of the aggressive form of tumor cells, the increased expression of Rap1GAP has been shown to associate with tumor suppression. The roles and functions of Rap1GAP can be tissue- or cancer-specific since some studies have shown that its overexpression may serve as a marker for tumor invasion. Therefore, further research is necessitated to uncover the roles of Rap1 and its GEFs and GAPs in carcinogenesis. Taken together, with all the results derived from current in vitro and in vivo studies, regulatory molecules of Rap1 signaling pathway could potentially be used as novel, reliable and rational biomarkers for cancer diagnosis and molecular targets for therapeutics. Further research is obligatory to uncover the downstream molecular targets and provide insights into signaling pathway of carcinogenesis in developing highly specific and novel targets for therapeutic interventions.

## Figures and Tables

**Table 1:** Roles of Rap1 in tumor progression.

Roles of Rap1	Tumor Types	Signaling Molecules	Results	Reference
**Tumor promoter**	Glioblastoma	Rap1A, thrombin, β1-integrin	Knockdown of Rap1A reduced more than 70% of tumor growth compared to control	[48]
	NSCLC	Rap1-GTP	Rap1-GTP depletion reduced growth of NSCLC cells and increased cisplatin sensitivity	[49]
	Melanoma	Rap1-GTP, p38	Rap1-GTP expression promoted melanoma migration and invasion	[50]
	Breast cancer	Rap1-GTP, β1-integrin	Pharmacological inhibition of Rap1-GTP and β1-integrin reduced cell migration in breast cancer	[51]
		Rap1	Expression of dominant-active Rap1 in breast epithelial cells increased invasiveness and tumorigenicity	[43]
	Prostate cancer	Rap1A, α4, β3 -integrins	Rap1A activation increased expression of α4- and β3-integrins, leading to increased tumor cell invasion	[26]
	Pancreatic cancer	Rap1-GTP, EGFR	Rap1-GTP activation promoted migration and EGFR-mediated metastasis of pancreatic cancer cells	[52]
**Tumor suppressor**	Bladder cancer	Rap1-GTP	Rap1-GTP activation suppressed migration of NBT-II bladder carcinoma cells	[53]

NSCLC, non-small-cell lung carcinoma; EGFR, epidermal growth factor receptor.

**Table 2:** Role of Rap1GAP in tumor progression.

Roles of Rap1	Tumor Types	Signaling Molecules	Results	Reference
**Tumor promotor**	HNSCC	Rap1, MMP9	Rap1GAP promoted SCC cell invasion via MMP9 production	[54]
	Acute myeloid leukemia	Rap1, MMP9	Overexpression of Rap1GAP promoted MMP9-mediated cell invasion	[55]
**Tumor suppressor**	Colorectal cancer	Rap1, MMP9, E-cadherin	Loss of Rap1GAP leading to tumor metastasis and poor prognosis	[56]
	Prostate cancer Melanoma	Rap1, integrin	Overexpression of Rap1GAP impaired tumor cell progression	[26]
	Melanoma	Rap1, ERK	Rap1GAP blocked Rap1 activation and inhibiting tumor proliferation and survival	[57]
	Endometrial cancer	Rap1, E-cadherin	Patients with higher expression of both Rap1GAP and E-cadherin had better survival	[58]
	Gastric cancer	Rap1, MMP2, E-cadherin	Rap1GAP increased E-cadherin and reduced cell migration and invasion	[46]
	Pancreatic cancer	Rap1, integrin	Overexpression of Rap1GAP blocked Rap1 activation and tumor invasion	[59]
	Oropharyngeal SCC	Rap1, ERK	Loss of Rap1GAP promoted Rap1 and ERK activation and tumor proliferation.	[47]

MMP, matrix metallopeptidase; ERK, extracellular signal-regulated kinase; FAK, focal adhesion kinase; HNSCC, head and neck squamous cell carcinoma; SCC, squamous cell carcinoma.
